# Draft Genome Sequences of Three Nitrogen-Fixing Strains Isolated from Soil Cooled for Growing Temperate Root Crops in a Tropical Climate

**DOI:** 10.1128/mra.00501-22

**Published:** 2022-08-24

**Authors:** Shazwana Shaárani, Nurul Syazwani Ahmad Sabri, Fatimah Azizah Riyadi, Fazrena Nadia Md Akhir, Nor’azizi Othman, Hirofumi Hara

**Affiliations:** a Department of Chemical and Environmental Engineering, Malaysia-Japan International Institute of Technology, Universiti Teknologi Malaysia, Kuala Lumpur, Malaysia; b Department of Mechanical Precision Engineering, Malaysia-Japan International Institute of Technology, Universiti Teknologi Malaysia, Kuala Lumpur, Malaysia; c Department of Biotechnology, Graduate School of Agricultural and Life Sciences, The University of Tokyo, Bunkyo-ku, Tokyo, Japan; University of Delaware

## Abstract

Nitrogen-fixing bacteria can form a symbiotic relationship with several members of the major plant groups and with fungi. Here, we present the draft genome sequences of three nitrogen-fixing strains isolated from soil cooled for growing temperate root crops in a tropical climate.

## ANNOUNCEMENT

Nitrogen-fixing bacteria are commonly found living freely in soil or aquatic habitats, or they form symbiotic relationships with plants (1–3). Previously, a novel strategy involving the use of a soil-cooling system (4) showed a significant effect of soil temperature on nutrient availability, especially nitrogen (4), with the cooled soil promoting better growth of root crops in a tropical climate. The increased nitrogen levels of the cooled soil were attributed to the presence of nitrogen-fixing bacteria, which requires further investigation. Here, we isolated and sequenced three different nitrogen-fixing bacterium strains, identified as *Agromyces* sp. strain C10, *Gordonia* sp. strain C13, and *Bacillus* sp. strain C21, from soil cooled for growing temperate root crops, to discover the mechanisms and enzymes that are responsible for promoting plant growth in tropical climates.

Soil samples were collected, following the harvest of temperate root crops grown in cooled soil, from a greenhouse at Universiti Teknologi Malaysia (UTM) (3°10.4836′N, 101°43.1933′E; Kuala Lumpur, Malaysia), as reported in our previous publication (4). A pure culture of diazotrophic bacteria was isolated following a modified protocol (5) in which bacteria were isolated by serial dilution and plated onto N-free (NF) medium containing sucrose as the carbon source. Axenicity of the cultures was confirmed by subculturing a single colony on an NF agar plate and observing the morphology of the isolated strain. Genomic DNA of the strains was extracted from single colonies of pure culture grown on agar plates, according to a modified protocol (6). A 350-bp library was constructed for sequencing using the NEB Ultra II library preparation kit according to the manufacturer’s protocol (NEB, Ipswich, MA). Sequencing was performed on a NovaSeq 6000 platform (Illumina, San Diego, CA), generating approximately 1 Gb of paired-end data (2 × 150 bp) for each sample. The raw Illumina paired-end reads were trimmed using fastp v0.21 (7) to remove low-quality bases and Illumina adapter sequences. Subsequently, the trimmed reads were used for *de novo* assembly in Unicycler v0.4.8 (8). Default parameters were used for the assembly. Contigs smaller than 500 bp, representing mostly sequencing artifacts, were removed, and the filtered assembly was used for subsequent analysis. Genome assembly statistics were generated using QUAST (9). rRNA-containing contigs were identified, and their corresponding rRNA gene sequences (5S, 16S, and 23S rRNAs) were extracted using Barrnap (https://github.com/tseemann/barrnap) into a single FASTA file; this file was then used to perform BLAST assessment against the GTDB (16S rRNA). The genomic features were then annotated using NCBI PGAP v5.1 (10), as shown in Table 1. According to the RAST v2.0 (11) annotation for each strain, numerous enzymes, including glutamine synthetase and glutamate synthetase, were predicted to be involved in nitrogen metabolism. Although these three isolates were able to fix nitrogen, no known nitrogen-fixing genes (*nifF*, *nifD*, *nifK, anf* and *vnf* complex, etc.) were found in the draft genome sequence. There might be other mechanisms involved in the nitrogen fixation of these isolates. Thus, understanding the genomes of these strains will provide insight into the plant-promoting mechanisms of bacteria.

**TABLE 1:** Genomic features of three nitrogen-fixing strains

Strain	Total no. of reads	Sequencing depth (×)	Genome size (bp)	No. of contigs	*N*_50_ (bp)	G+C content (%)	No. of CDSs^*a*^	No. of RNAs	No. of tRNAs	SRA accession no.
*Agromyces* sp. strain C10	742,916,100	226	3,285,654	19	239,959	72.15	3,091	3	44	SRR18827899
*Gordonia* sp. strain C13	757,767,000	148	5,109,131	74	137,589	67.38	4,500	3	49	SRR18827898
*Bacillus* sp. strain C21	534,232,350	132	4,039,111	24	1,046,335	43.69	4,011	4	55	SRR18827897

a CDSs, coding DNA sequences.

### Data availability.

The raw data for all three strains were deposited at GenBank under BioProject accession number PRJNA825673. The BioSample accession numbers for *Agromyces* sp. strain C10, *Gordonia* sp. strain C13, and *Bacillus* sp. strain C21 are SAMN27553938, SAMN27553939, and SAMN27553940, respectively. The GenBank accession numbers are JALPSX000000000 (*Agromyces* sp. strain C10), JALPSY000000000 (*Gordonia* sp. strain C13), and JALPSZ000000000 (*Bacillus* sp. strain C21). The SRA numbers are provided in Table 1.
